# Feasibility of the wingate anaerobic exercise test as a clinical measure in patients with juvenile dermatomyositis

**DOI:** 10.1186/s12969-022-00679-6

**Published:** 2022-03-28

**Authors:** Saunya Dover, Samantha Stephens, Hayyah Clairman, Andrew Abesamis, Omidali Aghababaei Jazi, Stephanie Babij, Jo-Anne Marcuz, Natasha Naraidoo, Jing Pan, Eleanor Pullenayegum, Dax Rumsey, Kristi Whitney, Brian M. Feldman

**Affiliations:** 1 Child Health Evaluative Sciences, The Hospital for Sick Children, Toronto, ON Canada; 2 Neurosciences and Mental Health, The Hospital for Sick Children, Toronto, ON Canada; 3 Division of Rheumatology, The Hospital for Sick Children, Toronto, ON Canada; 4 Department of Rehabilitation, The Hospital for Sick Children, Toronto, ON Canada; 5 Division of Rheumatology, University of Alberta, Edmonton, AB Canada; 6 Department of Pediatrics, Faculty of Medicine, University of Toronto, Toronto, ON Canada; 7 Institute of Health Policy, Management & Evaluation,, The Dalla Lana School of Public Health, University of Toronto, Toronto, ON Canada

**Keywords:** Juvenile dermatomyositis, Wingate anaerobic test, Feasibility

## Abstract

**Background:**

Core sets, while widely adopted for clinical assessment in juvenile dermatomyositis (JDM), have some drawbacks – they are time consuming, were developed primarily for research, and require an experienced multidisciplinary team. We propose the Wingate Anaerobic Test, a 30-s all out test performed on a cycle ergometer, as a potential alternative; it is valid and reliable in this patient population. We aimed to determine the feasibility of performing the Wingate test as part of a typical clinic visit, and to determine if it is correlated to current measures of disease activity.

**Methods:**

Patients 5–18 years of age, with JDM, were recruited from the JDM clinic at a large Canadian academic children’s hospital. Participants underwent a standard clinic assessment, then completed a Wingate test at the end of the visit.

**Results:**

Twenty-six patients participated in the study, representing a recruitment rate of 81%; of those, 88% were able to complete the Wingate test. Patients liked the Wingate test and felt it should be included as a regular clinic test. Absolute peak power (watts) on the Wingate test was strongly correlated to the manual muscle test (MMT-8) and the timed squat test. Relative peak power (watts/kg) on the Wingate test was strongly correlated to the timed squat test and the Childhood Myositis Assessment Scale (CMAS). Exploratory principal components analysis revealed that Wingate relative average power explained almost 2/3 of the variance of the CMAS, MMT and timed squats combined.

**Conclusion:**

The Wingate test is a feasible test for children with JDM and correlates well with standard clinical assessments. Given its brevity, it has the potential to replace more standard measures of physical function currently used in clinical assessments for children with JDM. Future work should focus on how best to operationalize Wingate testing in clinic without the use of dedicated personnel.

## Introduction

Children with juvenile dermatomyositis (JDM), a rare autoimmune disease characterized by muscle weakness and skin rashes, have a variable clinical course. With modern treatment options, the prognosis is generally quite good [1–3]. However, despite the availability of treatments, many patients suffer from chronic negative effects on health and fitness, such as poor endurance and fatigue [4, 5]. Compared to their healthy peers, children with JDM have lower functional capacity, even when the disease is inactive, which is in turn associated with a poorer health-related quality of life [6].

Accurate measures of disease activity and functional status in patients with JDM is important. Two international groups, the International Myositis Assessment & Clinical Studies group (IMACS) [7] and the Paediatric Rheumatology INternational Trials Organisation (PRINTO) [8] have endeavored to standardize the assessments of patients with JDM. The IMACS and PRINTO core sets consist of disease activity core set measures, disease damage core set measures, and patient reported outcomes [9]. These core sets are the current standard of care for assessing children with JDM and they are routinely collected in the JDM subspecialty clinic at The Hospital for Sick Children (SickKids) in Toronto, Canada.

Both the IMACS and the PRINTO core sets were developed primarily for research studies, and although they have been widely adopted clinically, they are not necessarily the most efficient or practical tools for use in a busy clinic setting.

Recently, an international consensus process defined an optimal dataset for use in clinical care of patients with JDM [10]. Clinical utility and efficiency were kept at the forefront during the development, and while this core set is certainly streamlined for clinical use, the process identified that more work is required to develop muscle assessment tools that are shorter and less redundant that current existing measures such as the Childhood Myositis Assessment Scale (CMAS) and Manual Muscle Testing (MMT).

The current core sets are time consuming. Preliminary data suggests the estimated time to complete them is up to 45 min, and could be even longer for less experienced clinicians [10]. The core sets are also relatively complex, and ideally require a multidisciplinary team consisting of at least a rheumatologist and a physical therapist.

A potential alternative test that may address several identified shortcomings of the core sets is the Wingate Anaerobic Test. The Wingate test is an exercise test that measures anaerobic capacity, and has been shown to be valid and reliable in children with JDM [5, 11]. During a Wingate test, the participant pedals against a constant resistance on a cycle ergometer as hard and as fast as possible for 30 s.

The Wingate test has several advantages. It is designed to be simple to administer without the need for personnel with advanced training, though some training in test administration and bike calibration/maintenance is required. It is relatively inexpensive, requiring only the one-time purchase of a cycle ergometer (the cycle ergometer we used [Monark Sport & Medical, Vansbro, Sweden] retails for approximately $8,000 USD), and software that is available free of charge from the cycle ergometer manufacturers, Monark Sport & Medical (Vansbro, Sweden; available from https://sport-medical.monarkexercise.se/software/). Testing is feasible over a large age span, making it an ideal test to follow patients throughout their lifespan [11]. Finally, it is a non-invasive, objective test that is a direct measure of muscle performance [12].

While potentially advantageous, the Wingate test may not capture all important aspects of JDM. JDM has both muscle and skin involvement [1–3] with the Wingate test only capable of assessing the former. Further, the Wingate test only measures anaerobic function (i.e., peak power, mean power and fatigue index) and does not look at other measures that may be important in the assessment of patients with JDM such as aerobic fitness, submaximal exercise capacity, and other system involvement [6].

The Wingate test was not included in the final core set of exercise studies for myositis [13] and to the best of our knowledge, the Wingate test has not been integrated into a JDM clinic setting. Previous studies in this patient population have been stand-alone work completed outside of routine clinical care [4, 5, 11, 14]. Therefore, the aims of the current study were to assess the feasibility of performing the Wingate test as part of a typical clinic visit in the JDM subspecialty clinic at SickKids, and to determine if the Wingate test is correlated to the other measures of disease activity collected during clinic assessments. We hypothesized that the Wingate test would have the following correlations with measures currently assessed in our clinic (detailed in methods): a moderate to strong positive correlation with the timed squats assessment, a moderate positive correlation with CMAS and MMT, a weak to moderate negative correlation with MYOACT-VAS and the Physician Global Assessment, a weak negative correlation with the CHAQ, Patient Global Assessment and nail-fold capillaroscopy, and a weak to very weak negative correlation with muscle enzyme levels.

## Methods

We conducted a cross sectional study where participants were asked to perform the Wingate test one time at the end of a single routine clinic visit. The SickKids Research Ethics Board approved the study, and all participants and/or parents/guardians provided written informed consent or assent.

We recruited a sample of consecutive patients from the JDM subspecialty clinic at SickKids who met the following inclusion criteria: a diagnosis of probable or definite JDM according to the Bohan and Peter criteria [15], age between 5–17 years, physically able to perform the Wingate test, and a minimum height of 132.5 cm (in order to fit on the cycle ergometer). Patients were excluded if they were unable to perform the Wingate test, at the discretion of the health care team (i.e., physically unable to perform the test, mentally incapable of understanding the test instructions, etc.).

Patients who participated in the study underwent a usual clinic assessment prior to completing the Wingate test. Upon arrival in clinic, the patient and/or their parent/guardian completed the Childhood Health Assessment Questionnaire (CHAQ) and the Quality of My Life (QoML) questionnaire. The CHAQ is a measure of functional status and gives a disability index score between 0–3, with 0 representing no functional disability and 3 representing maximal functional disability [16]. The QoML is a measure of both overall and health-related quality of life that uses 10 cm visual analog scales (VAS), where higher scores indicate better quality of life or health-related quality of life [17].

A physiotherapist (PT) or Advanced Practice PT completed several assessments of muscle strength and function. The Manual Muscle Testing (MMT-8) is a measure of muscle strength using a score of 0 (no contractions felt in the muscle) to 10 (holds test position against a strong pressure) for each of 8 muscle groups for a maximum score out of 80 [18]. The CMAS is a tool to assess physical function and is scored from 0–52, with 52 representing the highest possible level of physical function [19]. Finally, the patient performed a timed squat test, a legacy measure collected in our clinic, assessing the total number of bodyweight squats a patient can complete in 30 s.

Following the PT assessment, a clinic physician saw each patient and completed the following assessments: a physician global assessment of overall disease activity (scored from 0–10 with 10 representing maximal disease activity) [20], and the Myositis Disease Activity Assessment Visual Analogue Scales (MYOACT-VAS). The MYOACT-VAS is used to measure the degree of disease activity of both muscle and extra-muscular organ systems using a series of physician's assessments of disease activity of various organ systems. Each scale is scored from 0–10, with 0 representing no activity and 10 representing maximal activity for each area [21]. Muscle enzymes were measured and nail-fold capillaroscopy was assessed at all visits, with bloodwork being collected at the beginning of clinic prior to any other assessments [18].

At the end of all routine clinic assessments, patients completed the Wingate using a protocol that has been previously described and validated in this patient population [11]. The study coordinator administered the Wingate with assistance from research students; all received training from an exercise physiologist at SickKids. One tester was responsible for explaining the test to the participants and encouraging maximal effort while the other tester ran the computer software. Patients cycled against an external load calculated based on age and sex [12, 22]. Following a 3-min warmup and three 10-s sprints, we instructed the subjects to cycle as fast as they could against their pre-calculated load for 30 s. We recorded peak power, mean power, and end power to enable the calculation of a fatigue index (FI), which represents the decline in power over the 30 s test. Following the Wingate test, we had the subjects complete a short feasibility questionnaire.

Our primary outcome was the feasibility of using the Wingate test as a routine clinic measure. Our secondary outcome was the association of the Wingate test to the other measures routinely obtained during a clinic assessment.

We assessed the feasibility of the Wingate test using a combination of recruitment rate, proportion of completed Wingate tests, and the results of the feasibility questionnaire.

We summarized participant characteristics using descriptive statistics. We determined associations between the Wingate test and clinical measures using Pearson product-moment correlation coefficients or Spearman rank correlation coefficients depending on the distributions of the data.

As an exploratory analysis – to see whether the Wingate might be able to replace some of the time-consuming measures of physical function – we performed principal components analysis to determine the total variance explained by the CMAS, MMT-8 and timed squats and regressed the principal components against the Wingate test relative average power. This was done to see what proportion of the between subject variation could be accounted for by the Wingate test.

All analyses were completed using R version 3.6.2.^1^

We determined our sample size using a standard table of correlation coefficients. Our goal was to choose a sample size that would be likely to detect a moderate correlation between the performance on the Wingate test and our measures of disease activity in JDM (and differentiate it from a weak correlation). Strength of the correlations were interpreted according to the following definitions: ≤ 0.20 indicating very weak, > 0.2 to < 0.4 indicating weak, > 0.40 to ≤ 0.7 indicating moderate, > 0.70 to ≤ 0.90 indicating strong, and > 0.90 indicating very strong correlation. Therefore, we chose a sample size of a minimum of 25 [23].

## Results

Fifty-two patients were screened. Of those, 32 attended a clinic where study recruitment was taking place and were approached in clinic, and 26 (81%) consented to be enrolled into the study. Table 1 describes the clinical characteristics of our cohort.

**Table 1:** Demographics and Clinical Characteristics Demographics and clinical characteristics of the study cohort. All values are medians (interquartile range [IQR], range of values) unless otherwise indicated

	**Participants (*n* = 26)**
Female (n)	10 (38%)
Age, years	12.5 (3.8, 7–17)
BMI, kg/m^2^	20.1 (5.4, 14.2–34.2)
Age at diagnosis, years	7.0 (5.8, 0.75–12.0)
Years since diagnosis	3.0 (6.5, 0–14.0)
Medications^a^ (n)	
None	8 (31%)
Methotrexate	15 (58%)
Calcium/Vitamin D/Folic Acid	14 (54%)
Hydroxychloroquine	3 (12%)
IVIG	2 (8%)
MMF	1 (4%)
Other	6 (24%)
CHAQ Disability Index (0–3)	0 (0.125, 0–2.125)
Patient Global Assessment (0–10)	0.3 (1.3, 0–8.4)
Physician Global Assessment (0–10)	1.4 (3.9, 0–8.0)
Nailfold Capillaroscopy, n/mm, [mean (SD)]	5.5 (0.8)
CMAS (0–52)	52 (4.5, 27–52)
MMT-8 (0–80)	80 (7.8, 55–80)
Squat Test, n/30 s	22 (7, 9–30)
Wingate Test	
Peak Power (watts)	371 (204, 119–645)
Relative Peak Power (watts/kg)	6.2 (2.4, 4.2–9.7)
Fatigue Index (%)	50.4 (18.8, 33.6–102.6)
Average Power (watts/kg)	4.5 (2.2, 2.7–7.3)

^a^sum > 26 as subjects could be on more than 1 medication

*Abbreviations used*: *BMI* Body Mass Index, *IVIG* Intravenous Immune Globulin, *MMF* Mycophenolate Mofetil, *SD* standard deviation, *CHAQ* Childhood Health Assessment Questionnaire, *CMAS* Childhood Myositis Assessment Scale, *MMT-8* Manual Muscle Test

**Fig. 1:**
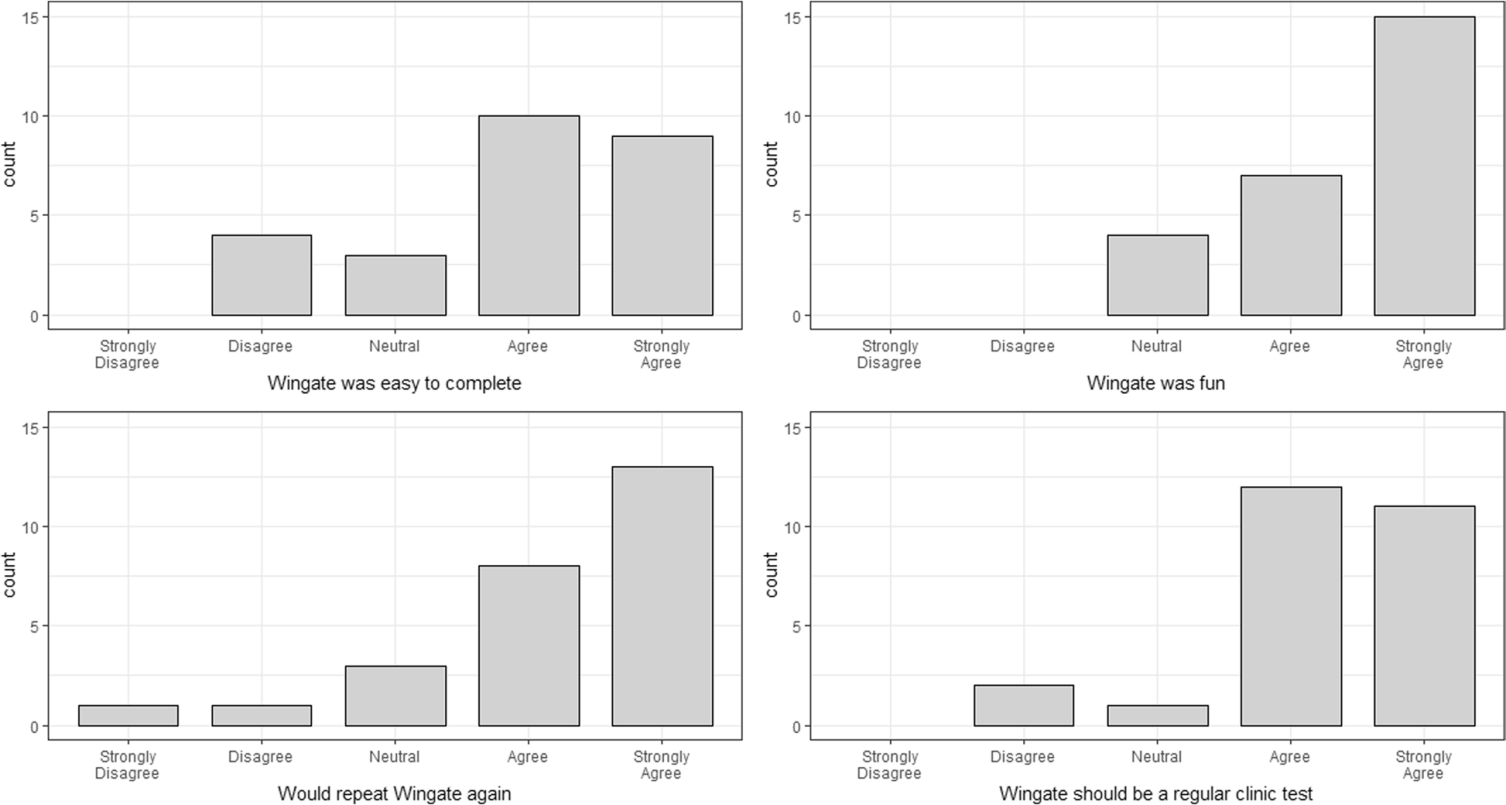
Distribution of levels of agreement from the Wingate feasibility questionnaire. Participants indicated their level of agreement with four statements immediately following their Wingate test: the Wingate was easy to complete, the Wingate was fun, I would do the Wingate again, and the Wingate should be a regular test as part of clinic

Of the 26 enrolled patients, 23 (88%) successfully completed the Wingate test. Of the three patients unable to complete the test, one was due to a software malfunction, and two were due to the patient being incapable of performing the test. Of those two, one child had never ridden a bicycle before, and one was physically capable but appeared unable to motivate themselves to complete the test, and both had inactive disease as determined by a physician global assessment score of 0. Overall, patients agreed that the Wingate was easy to complete, fun, should be a regular part of clinic and that they would do it again if asked (Fig. 1). Several patients found it difficult to complete but indicated that it was fun and that they would perform the test again if asked. Of those who did not find it easy nor fun, one subject indicated that they would repeat the test if the bike seat was more comfortable, and one subject indicated that it was difficult to walk after completing the test.

Absolute peak power (watts) on the Wingate test had a strong correlation with the MMT-8 and the 30 s squat test, and a moderate correlation with the CMAS (Table 2).

**Table 2:** Association of Wingate outcomes to clinical parameters

Variable	Absolute Peak Power (Watts)		Relative Peak Power (Watts/kg)		Fatigue Index	
	**r**	***p*-value**	**r**	***p*-value**	**r**	***p*-value**
CHAQ	-0.38	0.074	-0.65	0.0009	-0.15	0.51
Patient Global Assessment	-0.38	0.071	-0.53	0.01	0.00	0.99
Physician Global Assessment	-0.58	0.004	-0.60	0.002	-0.10	0.65
Nail fold capillaroscopy	0.32^a^	0.14	0.32^a^	0.14	-0.17^a^	0.44
MYOACT-VAS^b^						
Constitutional Disease Activity	-0.17	0.45	-0.44	0.036	-0.03	0.90
Cutaneous Disease Activity	-0.59	0.003	-0.54	0.008	-0.08	0.73
Skeletal Disease Activity	-0.49	0.017	-0.42	0.049	-0.06	0.77
Extra-muscular Global Assessment	-0.64	0.001	-0.57	0.004	-0.10	0.66
Muscle Disease Activity	-0.46	0.028	-0.63	0.001	0.00	0.99
Global Disease Activity	-0.62	0.002	-0.60	0.003	-0.05	0.81
ALT	-0.33	0.12	-0.41	0.051	-0.28	0.19
AST	-0.36	0.096	-0.44	0.034	-0.01	0.95
CK	-0.091	0.68	0.028	0.90	-0.26	0.24
LDH	-0.24	0.26	-0.33	0.12	-0.31	0.15
CMAS	0.59	0.003	0.78	< 0.0001	-0.07	0.76
MMT-8	0.71	< 0.0001	0.69	0.0002	0.22	0.32
Timed Squats	0.69	0.001	0.79	< 0.0001	-0.06	0.82

^a^Pearson’s product moment correlation coefficient

^b^Gastrointestinal disease activity, pulmonary disease activity, cardiovascular disease activity, and other disease activity are not reported as all scores were 0

Correlations between absolute peak power, relative peak power, and fatigue index, and clinical assessments. All *r*-values are Spearman’s rank correlation coefficients unless otherwise indicated. *Abbreviations used*: *MYOACT-VAS* Myositis Disease Activity Assessment Visual Analogue Scales, *ALT* Alanine Aminotransferase, *AST* Aspartate aminotransferase, *CK* creatine kinase, *LDH* lactate dehydrogenase, *CMAS* Childhood Myositis Assessment Scale, *MMT-8* Manual Muscle Test

Relative peak power (watts/kg) on the Wingate test had a strong correlation with the 30 s squat test and the CMAS, a moderate to strong correlation with the MMT-8, and a moderate correlation with AST (Table 2).

There were no significant correlations between the fatigue index and any of the clinical measures. Table 2 shows a complete list of our outcome measures.

The CMAS, MMT and time squats were highly correlated. Principal components analysis showed that a single first factor, that loaded on all 3 variables, explained 81% of the variance among them. Together, the CMAS, MMT-8 and timed squats characterize between subject variation; the Wingate relative average power explained almost 2/3 of this variance (R-squared = 64.7%, F_3,14_ = 8.54, p < 0.002) (Table 3).

**Table 3:** Principal Components Analysis

	**PC1**	**PC2**	**PC3**
EigenValue	2.43	0.42	0.15
% total variance	81.0%	13.7%	5.1%
EigenVectors			
CMAS	-0.610	-0.009	-0.793
MMT-8	-0.560	0.713	0.422
Timed squats	-0.561	-0.701	0.440

Principal components analysis showing the EigenValues, total variance explained, and factor loadings (EigenVectors) of each principal component (PC). Of the 26 subjects, 5 were incomplete (3 missing Wingate, 2 missing timed squats). *Abbreviations*: *CMAS* Childhood Myositis Assessment Scale, *MMT-8* Manual Muscle Test

## Discussion

The Wingate test appears to be a feasible addition to a typical visit in a JDM clinic. Positive feedback from patients supports the feasibility of incorporating the test into their standard clinical assessment. The Wingate test was also significantly correlated with many of the more time-consuming clinical outcomes collected during clinic visits, especially the CMAS and MMT-8 and the 30 s squat test; this suggests that it is a good measure of muscle function in this population.

We have shown that the Wingate test is a feasible test for this patient population. The combination of ease of recruitment, high completion rates, and positive feedback suggest that this test could be used during the regular clinical follow up of patients with JDM. While the majority of our cohort liked the test, it would be worth exploring the possibility of a more comfortable seat for the cycle ergometer if the Wingate were to be administered on a consistent basis.

The Wingate test also has good reliability as a test for children with JDM [11]. In the present study, we were able to successfully administer the Wingate in children as young as 7 years of age. Takken and colleagues have previously demonstrated that the Wingate test is reliable in children and young adults with JDM with a mean age of 13.85 (range 6.7–27.2) [11]. Furthermore, patients with a range of disease activity, from clinically inactive to flaring, were able to complete the test, as evidenced by the range of disease activity scores in our cohort and that the only participants unable to complete the test had inactive disease. Taken together, these factors suggest that the Wingate may be an appropriate measure to be included in any core set of measures of exercise tolerance and/or physical function in children with JDM.

In our exploratory principal components analysis, we showed that Wingate relative average power explains almost 2/3 of the variance of the CMAS, MMT-8 and timed squats combined. This suggests that these assessments are all measuring the same underlying construct, and that perhaps the Wingate may be able to replace one, or more, of these measures. Some of these assessments, especially the CMAS and MMT-8, are time consuming, require expert judgement, and are limited in their usefulness by ceiling effects; for example in our cohort, almost 60% of patients achieved the highest score possible on the CMAS. With these known ceiling effects on the CMAS, it is possible for a more muscular teen to be experiencing weakness relative to their baseline but still achieve the highest score possible. The Wingate test is an objective measure of muscle function, which is not bound by ceiling effects. It is also relatively quick to complete. The recovery time for each participant was variable, but that time could pass while other clinic activities were being carried out.

While promising, our study results should be interpreted given some limitations. The testing was done with a relatively small sample at a single centre in a group of children with relatively good physical function, as evidenced by the median CMAS and MMT scores of our cohort being the maximum values provided by these tests. We did have several patients in our cohort with higher levels of disease activity (i.e., a CMAS score < 35) who were able to complete the test and who provided positive feedback that it was fun. While our high test completion rate, regardless of level of disease activity, and positive feedback from patients suggest that our results would be generalizable, further testing on larger cohorts, including patients with more severe disease activity, should be carried out.

During this study, the Wingate testing was conducted by designated research personnel in a clinic room that was reserved for research. Adding on the test to the current clinical protocol would require resources, such as a designated space large enough to perform the test, funding for a cycle ergometer with a computer, and appropriately trained personnel to conduct and supervise the testing. If, however, the Wingate test was used to replace time-intensive assessments (e.g., CMAS, MMT-8, timed squats), it might be resource saving.

Finally, a criticism of the Wingate test is that the outcome can be influenced by the participant’s motivation to perform the test [11] as well as their understanding and/or comfort with committing a maximal effort. In order to try to minimize the effects of motivation, the test administrators were consistent and testing procedures were standardized. Variations in motivation, of course, influence the other measures of physical function (like the CMAS, MMT-8 and timed squats) in a similar way.

Overall, we have shown that the Wingate test is a feasible test for children with JDM, and that it correlates well with clinical assessments. We have also shown that it has the potential to replace one or more measures of physical function currently used in JDM clinic assessments. Additional work should be done to confirm these results, to determine if a similar correlation exists between the Wingate test and the standard myositis assessments in newly diagnosed patients, and to determine how best to operationalize the testing using only clinical personnel. Longitudinal studies with repeated Wingate testing would also be of benefit to determine if the Wingate test could help to predict disease flares.

## Data Availability

The datasets used and/or analysed during the current study are available from the corresponding author on reasonable request.

## Footnotes

1 R Core Team (2019). R: A language and environment for statistical computing. R Foundation for Statistical Computing, Vienna, Austria. URL https://www.R-project.org/
